# A Rare Case of Aeromonas Hydrophila Infection in a Patient With Hereditary Hemochromatosis

**DOI:** 10.7759/cureus.20612

**Published:** 2021-12-22

**Authors:** Adham E Obeidat, Linda L Wong, Larissa Fujii-Lau

**Affiliations:** 1 Internal Medicine, University of Hawaii, Honolulu, USA; 2 Surgery, University of Hawaii, Honolulu, USA; 3 Gastroenterology and Hepatology, The Queen's Medical Center, Honolulu, USA

**Keywords:** pancreatico-duodenectomy, sepsis, liver, pancreatic cancer, hereditary hemochromatosis

## Abstract

Hereditary hemochromatosis (HH) is a genetic disorder characterized by abnormal iron metabolism, which leads to elevated serum iron levels and iron tissue deposition. This can suppress immunity and increase pathogen virulence, increasing the susceptibility to serious infections. We present a case of a 76-year-old man with a history of HH, who was later found to have pancreatic adenocarcinoma. He underwent a pancreaticoduodenectomy that was complicated by an unusual *Aeromonas hydrophila *septicemia that leads to death. It is important for physicians to appreciate the potential for highly unusual and life-threatening infections in the management of patients with HH.

## Introduction

Hereditary hemochromatosis (HH) is an autosomal recessive disorder caused by mutations in the high Fe (HFE) gene, most commonly (90%) due to homozygosity for C282Y [1]. Mutations in the HFE gene lead to excess iron absorption in the form of non-transferrin-bound iron, which causes injury and is readily taken up by cardiomyocytes, pancreatic islet cells, and hepatocytes [2]. Although a rare condition (affects one in 300-500 individuals), HH is considered one of the most common genetic disorders in white people [1,2]. Since not all patients with HFE mutations have iron overload, genetic and environmental factors, dietary iron intake, and blood loss contribute to the accumulation of iron over time.

Most symptomatic patients present at an older age with non-specific signs and symptoms such as fatigue and arthralgia [2,3]. Impaired iron metabolism has been implicated in the increased susceptibility to various and unusual bacterial infections and septicemia [4]. *Aeromonas hydrophila* is a gram-negative rod that usually causes mild to moderate infections. However, it can be associated with fatal complications in immunocompromised individuals. We present a case of a fatal*A. hydrophila* septicemia in a patient with HH who underwent pancreaticoduodenectomy for early pancreatic cancer.

This case was submitted as an abstract at the American College of Gastroenterology Annual Scientific Meeting, Las Vegas/Nevada, October 22-27, 2021.

## Case presentation

**Figure 1:**
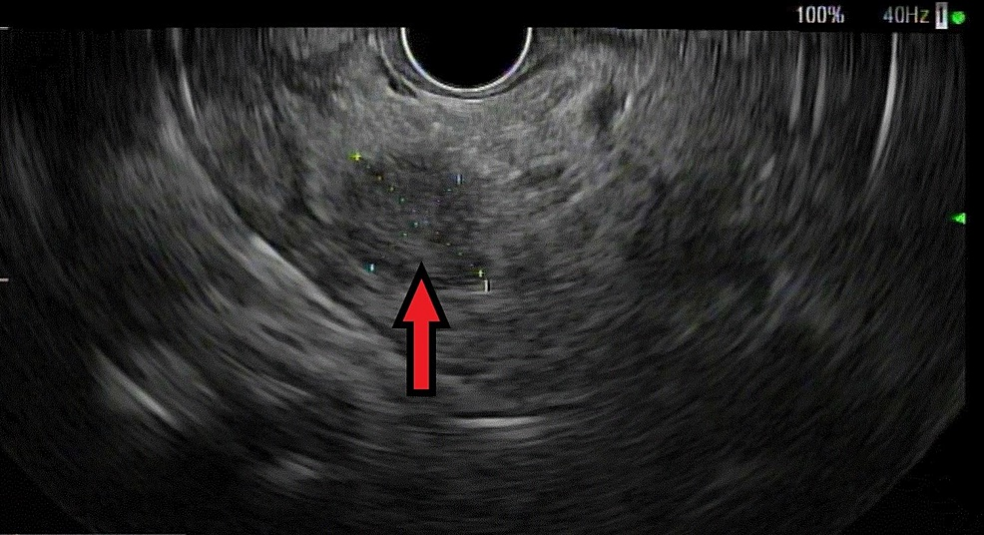
Endoscopic ultrasound image (red arrow) shows a 15 mm mass in the head of the pancreas with no vascular invasion or surrounding pathological lymph nodes.

**Figure 2:**
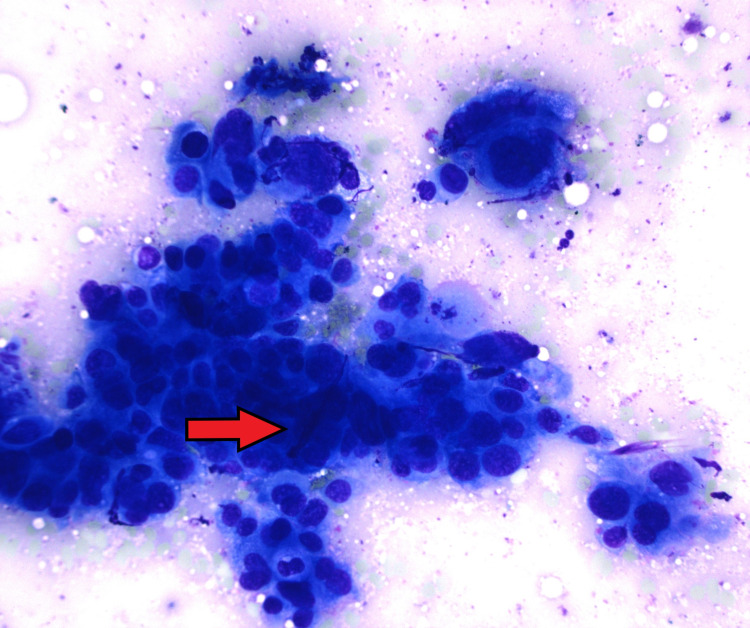
Cytologic smear (red arrow) shows crowded clusters of malignant cells with marked pleomorphism and hyperchromatism consistent with pancreatic ductal adenocarcinoma.

A 76-year-old man with remote follicular lymphoma treated with chemoradiation, recurrent small bowel obstructions, and hypertension underwent a liver magnetic resonance image (MRI) for persistently elevated ferritin and iron saturation that did not decrease despite cessation of his iron supplementation. The MRI confirmed iron overload in the liver and spleen, and incidentally found a double duct sign with a dilated bile and pancreatic duct without an obvious mass. His oncologist diagnosed him with HFE-related HH through genetic testing, started him on monthly phlebotomy and referred him for an endoscopic ultrasound (EUS). The EUS revealed a 15 mm mass in the pancreatic head with no vascular involvement or pathologic-appearing lymph nodes (Figure 1). Fine needle aspiration confirmed pancreatic adenocarcinoma (Figure 2). After staging computed tomography (CT) scans, the patient was diagnosed with T1N0Mx pancreatic adenocarcinoma. Neoadjuvant chemotherapy was declined by the patient.

The patient underwent exploratory laparotomy with lysis of adhesions, pancreaticoduodenectomy, and cholecystectomy. The tumor was focally adherent to the superior mesenteric vein. He had no intraoperative complications. The final stage of his malignancy was pT2N1 with one out of seven regional lymph nodes involved with metastatic disease. The patient recovered well until the late post-operative day (POD) one, when he developed hypotension and tachycardia. He received multiple fluid boluses, and hemoglobin decreased from 8.6 to 5.0 g/dL in seven hours. Despite the acute anemia, he had no clinical evidence of bleeding with no abdominal distension or blood from his surgical drain. Laboratory tests were significant for a slightly elevated white blood cell count (WBC) of 11.3 × 10^9^/L, platelet count of 108 × 10^3^/uL, acute kidney injury with a serum creatinine of 1.7 mg/dL from a baseline of 0.7 mg/dL on admission, mildly elevated aspartate aminotransferase (AST) at 94 units/L, total bilirubin level of 1.7 mg/dL, and an elevated lactate at 11.1 mmol/L and procalcitonin 41.43 ng/mL.

The patient was transferred to the surgical intensive care unit for further management and was started on vasopressors. He received two units of packed red blood cells (PRBCs) and hemoglobin increased to 8 g/dL. Shortly after this, he developed a mottled necrotizing soft tissue infection in his abdominal wall. Blood cultures grew *A. hydrophila*. Subsequently, he was started on meropenem and ciprofloxacin empirically. Despite supportive management, his condition continued to deteriorate and he required mechanical ventilation. The *A. hydrophila* strain was found to be carbapenem-resistant, therefore, his antibiotic coverage was changed to ceftazidime-avibactam, vancomycin, and metronidazole. Unfortunately, the patient’s condition continued to deteriorate and he eventually expired due to irreversible septic shock on POD seven.

## Discussion

Although HH is a rare condition, it remains the most identifiable genetic disorder in individuals of European ancestry [1,2]. The main gene defect alters the expression of the HFE protein responsible for regulating hepcidin. Hepcidin decreases intestinal iron absorption by enterocytes and decreases iron release by macrophages in response to excess plasma iron. Altered HFE protein will lead to decreased hepcidin expression and therefore, unregulated iron levels [5].

Excess body iron can increase pathogen virulence via different proposed mechanisms [6,7]. One mechanism suggests that high transferrin saturation compromises bacteriostatic properties, and the pathogen’s ability to procure iron from transferrin will increase [7]. Moreover, iron overload can compromise the function of phagocytic cells through the formation of excess oxygen radicals, this alters phagocytosis through the peroxidation of neutrophil membrane lipids [7,8]. Furthermore, inadequate expression of hepcidin in patients with functional impairment due to high iron concentrations might increase susceptibility to infections [9].

Iron overload also increases patient susceptibility to invasive bacterial infections (including *Vibrio vulnificus*, *Vibrio cholerae*, *Escherichia coli*, *Yersinia enterocolitica*, and *Listeria monocytogenes*), viruses, as well as fungi [6,7]. However, previous cases of *A. hydrophila* infection associated with HH have not been reported.

*A. hydrophila* is a gram-negative rod, found mainly in fresh and saltwater, domestic and hospital water supplies, and food such as fish and shellfish. It is acquired by ingesting contaminated food or through direct contact with contaminated water supplies [10,11]. Typically, *A. hydrophila *infection presents as gastroenteritis or mild to moderate soft tissue infection. However, in immunocompromised individuals and patients with chronic diseases, such as liver cirrhosis, it can cause more severe and fatal infections [11,12]. We think that his prior chemoradiation therapy and partial small bowel resections may have exposed him to *A. hydrophila*, while his underlying HH predisposed him to a more severe and ultimately fatal infection. *A. hydrophila* infection has been reported to be complicating microsurgical procedures and leech treatment [13].

Most *Aeromonas* strains are resistant to penicillin and ampicillin but are susceptible to trimethoprim-sulfamethoxazole, fluoroquinolones, cephalosporins, aminoglycosides, carbapenems, and tetracyclines [14]. However, there have been several reports of infection with fluoroquinolone-resistant *A. hydrophila* strains [15]. Therefore, species identification and susceptibility testing for all isolates are crucial, and fluoroquinolones should potentially be avoided in the setting of serious infection.

## Conclusions

Finally, physicians should be aware of the potential risk of infection in patients with HH even when undergoing routine procedures. HH may not only increase the risk of unusual infections but also the severity of the infection, so looking purposely for such infections should be considered in these patients in order to give the proper management on time.
